# A Biophysical Model for the Staircase Geometry of Stereocilia

**DOI:** 10.1371/journal.pone.0127926

**Published:** 2015-07-24

**Authors:** Gilad Orly, Uri Manor, Nir S. Gov

**Affiliations:** 1 Department of Chemical Physics, The Weizmann Institute of Science, P.O.B. 26, Rehovot, Israel 76100; 2 Section on Organelle Biology, 35 Convent Drive, Porter Neuroscience II, NIH, Bethesda, Md. 20892, United States; University of Heidelberg Medical School, GERMANY

## Abstract

Cochlear hair cell bundles, made up of 10s to 100s of individual stereocilia, are essential for hearing, and even relatively minor structural changes, due to mutations or injuries, can result in total deafness. Consistent with its specialized role, the staircase geometry (SCG) of hair cell bundles presents one of the most striking, intricate, and precise organizations of actin-based cellular shapes. Composed of rows of actin-filled stereocilia with increasing lengths, the hair cell’s staircase-shaped bundle is formed from a progenitor field of smaller, thinner, and uniformly spaced microvilli with relatively invariant lengths. While recent genetic studies have provided a significant increase in information on the multitude of stereocilia protein components, there is currently no model that integrates the basic physical forces and biochemical processes necessary to explain the emergence of the SCG. We propose such a model derived from the biophysical and biochemical characteristics of actin-based protrusions. We demonstrate that polarization of the cell’s apical surface, due to the lateral polarization of the entire epithelial layer, plays a key role in promoting SCG formation. Furthermore, our model explains many distinct features of the manifestations of SCG in different species and in the presence of various deafness-associated mutations.

## Introduction

Stereocilia are actin-based membrane protrusions that are bundled together to compose the mechanosensitive organelle of auditory and vestibular hair cells [1]. The stereocilia staircase geometry (SCG) is an extremely complicated structure maintained by dozens of different proteins. In this paper we wish to advance the understanding of this complex problem using a simplified theoretical approach [2] that accounts for physical forces and fluxes that influence actin protrusion geometry in general [3–5], and stereocilia in particular [6, 7].

Let us review briefly the key properties of the stereocilia, which are essential for the remainder of this paper. Mature stereocilia are organized in rows of graded lengths, with a staircase geometry (SCG), across the apical surface of hair cells. Each stereocilium is ∼ 0.2 − 1*μm* in diameter and 1 − 10’s *μm* in length, composed of hundreds of tightly crossed linked parallel and uniformly polarized actin filaments [8], packed in a cylindrical bundle that tapers at the base. Various proteins including myosin motors and actin polymerization regulators work to maintain the stereocilia’s precise shape and functionality over the organism’s lifetime. The functionality of the hair cell is dependent on a direct relationship between the spatial deflection of the stereocilia and the influx of Ca^2+^ and K^+^ ions into the cell [9, 10]. Stereocilia of consecutive rows are connected by heterotypic dimers of cadherins CDH23 and PCDH15, which form extracellular links (tip-links). When the stereocilia staircase deflects, the tip-links pull open ion channels at the tips of the shorter rows of stereocilia, thereby depolarizing the hair cell.

At the base of the stereocilia the actin bundle tapers and extends as a rootlet, elongating 1 − 2*μm* into the cell’s cuticular plate—a dense mesh composed mainly of a network of actin filaments. Although the rootlet is a direct continuation of the actin bundle in the protrusion, it is more densely packed than the protrusion and includes distinct bundling proteins [11, 12]. The stereocilia polymerization rate is about 1000 fold smaller than the typical rate in filopodia, and in mammalians is proportional to the stereocilia’s height [13] (with longer protrusions having faster polymerization rate). As the stereocilia form and elongate, the expression levels of some of the different proteins change, either increasing or decreasing until reaching their final levels (e.g. [14, 15]). While many SCG are relatively simple, with incremental changes in length and thickness for each row, other geometries, such as the cochlear inner hair cell, are more complex, manifesting non-linear changes in both thickness and length from the shortest to the tallest row. The complexity of stereocilia, together with the notorious difficulty of experimenting with hair cells, makes them extremely challenging to study, both theoretically and experimentally. Therefore an understanding of the roles of all the different proteins and their interactions, let alone a comprehensive theory of the formation of this system, is still lacking. Despite a very recent attempt to to provide a quantitative model to account for some aspect of the simple SCG [16], there is currently no proposed model that can account for the variety of observed stereocilia bundles in different hair-cells, different species, and in the presence of mutations.

We propose a model that is focused on the actin and the regulating proteins dynamics that determine, by a balance of forces and fluxes, the height and width of stereocilia. We apply our recently published theoretical model [2] for the shape of actin-based cellular protrusions, which combines biochemical and physical processes, in order to explain the complex structure and dynamics of the SCG in normal cells and in the presence of different mutations. One common conclusion from both [16] and our model is that the formation of the SCG should involve the existence of an intracellular gradient along the apical surface. Our model connects this gradient to a quantitative analysis of the interplay between actin dynamics and membrane forces. We demonstrate that our proposed model explains many of the puzzling and sometimes seemingly contradictory observations in a unifying way.

## Model

We apply here our theoretical model [2] to describe the SCG of stereocilia, and refer the reader to [2] for more details. The model for the geometry of actin-based protrusions is composed of two parts, the first describes the protrusion’s height dynamics and steady-state (St.St) solution, in terms of the balance between the restoring and the pushing forces. The restoring force is applied mainly by the membrane elasticity, and by molecular motors that connect the actin bundle to the membrane (e.g. myosin I, myosin VII). The protrusive force is due to the treadmilling velocity of the actin pushing the rootlet inside the viscous-like cytoplasmic medium. The cytoplasmic network providing the friction and support of the stereocilia undergoes remodelling such that over long time-scales stereocilia dynamics can be treated as effectively viscous.

These forces, and therefore also the protrusion height, are strongly affected by the polymerization rate which is determined to a large extent by the concentration and activity of different actin regulating proteins at the protrusion’s tip and their transport mechanism (i.e. free diffusion and active transport by different myosin motors), the concentration profile of severing proteins along the bundle, and the concentration of actin-membrane myosin connectors. The height dynamics are described by the following equation (all the symbols are presented in Table 1):

$$\dot{h}=\frac{\gamma_{c}S_{c}\left[h\left(t\right),t\right]A\left[h\left(t\right)\right]+F_{ma}\left[h\left(t\right)\right]+F_{md}\left[h\left(t\right)\right]}{\gamma_{c}S_{c}\left[h\left(t\right),t\right]+\mu }$$ (1)

while the St.St height of a cylindrical protrusion is given by solving the equation:

$$h_{st}=\frac{\gamma_{c}}{\alpha }R_{tip}A\sqrt{1+{\left(A/\beta \right)}^{2}}$$ (2)

where both the actin polymerization rate *A* and the radius at the protrusion tip (“tip-complex”) *R*_*tip*_ depend on the height.

**Table 1:** List of the variables and parameters used in Eqs 1 and 2.

Symbol	Meaning
*h*(*t*)	protrusion height
*z*	coordinate along the protrusion
*γ*_*c*_	cytoplasm effective viscosity coefficient
*α*	average restoring force of single actin-membrane myosin connector
*μ*	effective friction coefficient between the membrane and the cytoplasm around the protrusion
*β*(*z*)	severing rate profile along the protrusion
*A*(*h*)	polymerization rate
*S*_*c*_[*h*, *A*(*h*), *β*(*z*)]	rootlet surface area
*F*_*ma*_[*h*(*t*)]	total restoring force of the actin-membrane myosin connector along the protrusion
*F*_*md*_[*h*(*t*)]	restoring force due to the bending of the membrane around the actin bundle
*R*_*tip*_(*t*)	the actin bundle radius at the protrusion’s tip

The strength of the model is that it facilitates association between the general biochemical properties of the different components and the protrusion’s height. The key conclusions that arise from the model are: (i) St.St height is maintained by the restoring force of actin-membrane myosin connectors in the regime where the polymerization rate increases with the protrusion height (as observed in stereocilia [13]), (ii) the effective viscosity of the underlying cytoplasm affects the St.St height and, (iii) the possibility for multiple St.St height solutions for a single isolated protrusion, even in a spatially uniform cell.

The second part of the model [2] deals with the protrusion radius. It shows that the radius of the tip-complex can be dynamically regulated and is determined by the concentration of F-actin nucleators, G-actin, actin cross-linkers (CL) and nucleator deactivators at the tip complex. The final St.St radius of the tip-complex depends on the properties, transport mechanism, and reaction rates of these components. The main results from this model are: (i) the tip radius depends on the polymerization rate as well as on the height of the protrusion, such that (ii) the protrusion radius can either shrink or expand as the protrusion height increases (depending on the characteristics of the transportation mechanism of the proteins that affect the polymerization), and (iii) there is a minimal height below which the protrusion radius falls to zero (a stable tip complex cannot be maintained). We emphasize that both parts of the model are in a form of functional relations between the different components, which themselves may have different functional forms (e.g. the dependence of the polymerization rate *A* on the height in Eqs 1 and 2). These functions can be derived from the specific biochemical and physical processes in the cell. Therefore according to the model while the underlying mechanisms that control the protrusions’ dynamics are the same in all or most cell types, the specific characteristics depend on the exact protein compositions. The model we present should therefore be regarded as a general framework for the analysis of the possible mechanisms that control the stereocilia SCG. The proposed model aims to explain puzzling observations regarding the stereocilia SCG, the relation between the stereocilia and the microvilli (MV) that precede it, and provide predictions that can be checked for further verification.

## Results

The basic observation that the apical surface of the hair cell can give rise to stable stereocilia of different St.St height (and radius), can be explained by either (i) multi-stability of a uniform cell or (ii) due to spatial inhomogeneity. We analyze both options below, and conclude that the second possibility is more likely. The dynamics of stereocilia formation is discussed in S1 Text.

### Multi-stability in a uniform cell

**Fig 1:**
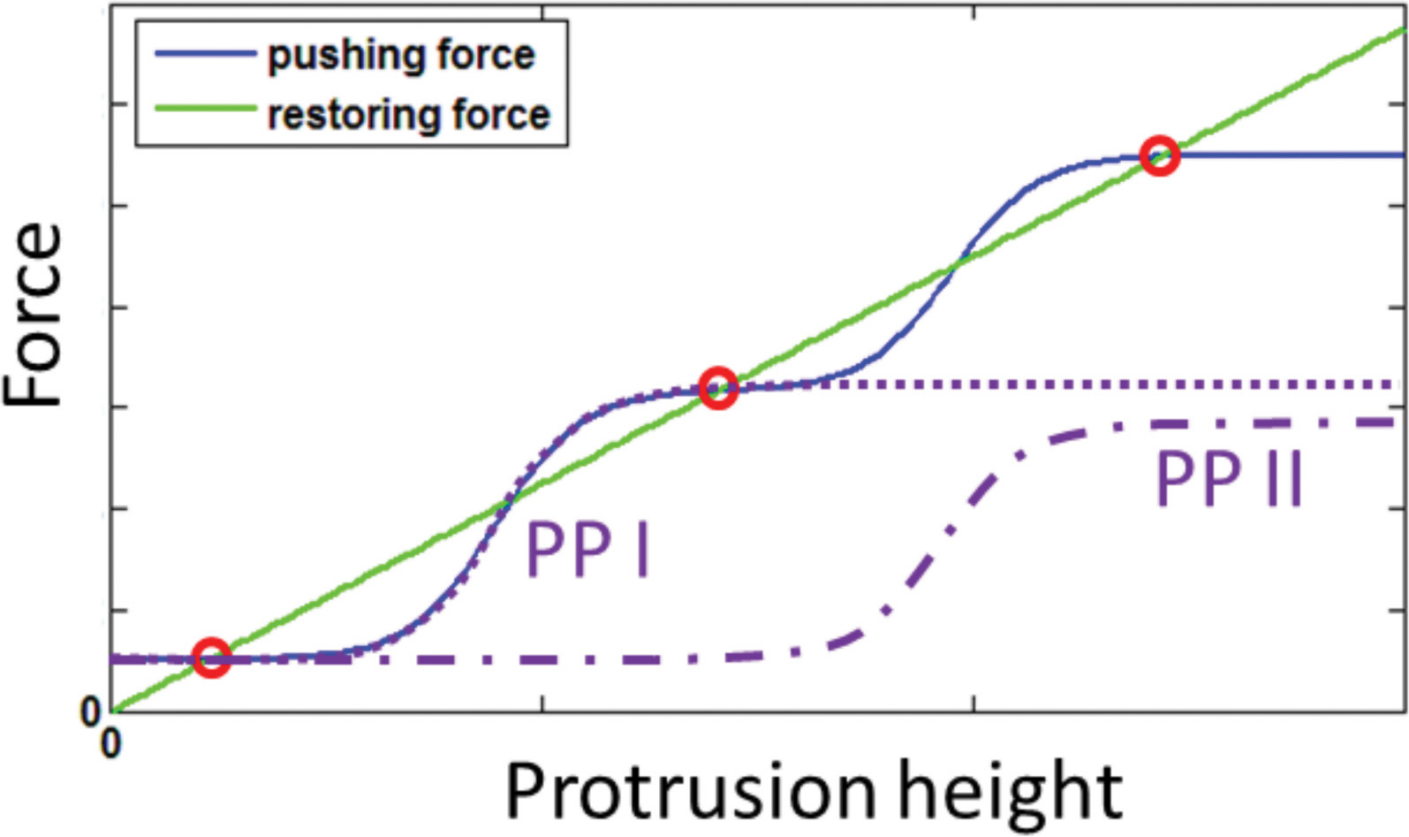
The pushing force resulting from two different promoters of actin polymerization, with different saturation heights, may result in multiple steady-state solutions. The purple dash-dot and dotted lines are the concentration profiles of the two promoters at the tip. The blue line is the pushing force due to the polymerization rate determined by the promoter concentrations at the tip, and the green line is the restoring force, which is dominated by actin-membrane myosin connectors. The red circles mark the stable steady-state heights, *h*_*st*_ (Eq 2).

The graded heights within the stereocilia bundle, could result from the appearance of multiple St.St heights in a spatially uniform cell. Such solutions are possible when the polymerization rate *A*(*h*) itself has a staircase-like dependency on the height [2]. This can be achieved when *A*(*h*) is determined by several different species of promoters of actin-polymerization with height-dependent concentrations [6]. According to our model of actin-based protrusions [2] the pushing force in a cylindrical protrusion has the same height dependency as *A*(*h*) (Eq 2), while the restoring force is dominated by the membrane-actin myosin connectors and increases with stereocilia height (*F*_*ma*_). Fig 1 demonstrate such multiple St.St solutions using two different types of promoters, each being transported to the tip by myosin motors but saturating at different heights. The lateral organization of the stereocilia of different heights into a SCG could then be driven by the maximization of inter-stereocilia links, around the kinocilium (Figure A in S1 Text).

While this scenario is theoretically possible, and allows great flexibility to the system, this solution suffers from two main limitations: (i) For each additional row of stereocilia there is a need for additional species of actin promoter, whereas hair cells of less developed organisms have a simpler protein network yet include more rows than hair cells of mammals [17]. (ii) Fluctuation in the concentration levels of proteins would result in a complete loss of some of the solutions, resulting in the loss of rows. This is not observed, suggesting that either there is strong feedback regulation in this system to maintain the solutions, or that this is not the correct mechanism for the SCG.

Another possible mechanism that may affect the heights is related to the spontaneous oscillations of the stereocilia [18, 19]. As the stereocilia oscillate the angles between the stereocilia and their roots vary between rows. The tip-links between the rows form an angle gradient which could also influence the pushing force and therefore shift the St.St height solutions between the rows, giving rise to the SCG. This mechanism presents an appealing notion whereby the stereocilia functionality, as exhibited by their spontaneous oscillations, self-organizes the structure (SCG) which is essential for their function. While this is an elegant mechanism, there is evidence that the SCG forms even when the tip-links are removed [20], although the stereocilia in this case eventually decay. Furthermore, the spontaneous oscillations are observed at a later time in the hair-cell development, after the SCG has already clearly began to form [21]. We therefore conclude that while this feedback mechanism might take part in the adjustment of the stereocilia heights to the oscillation resonance along the cochlea, it is not the main origin of the SCG.

### Non-uniform cell

From the discussion above, it can be concluded that a uniform cell is less likely to support the formation of the SCG with several rows. We therefore propose that there are spatially non-uniform properties along the apical surface of the hair cell that directs the gradient in the heights. There are several observations to support this hypothesis: Membrane-bound signaling molecules produce an overall planar polarization of inner ear tissue [22]. This planar polarization is manifested as an asymmetry within the cell, very similar to gradients of morphogens in multi-cellular tissues. The existence of the kinocilium is another source of non-uniformity in the cytoskeleton of the apical surface of the hair cells. Furthermore, a gradient in the cuticular plate mechanical properties (viscosity *γ*_*c*_) is indicated by the observed structural non-uniformity of the cuticular plate [11].

The main properties that control the stereocilia height are (Eq 2) the rate of polymerization (*A*), the actin severing rate at the rootlet and base (*β*) and the effective viscosity of the cytoplasm (*γ*_*c*_). For simplicity let us consider that there is a linear spatial gradient in *γ*_*c*_ (although similar results arise from a spatial gradient in *A* or *β*). The initial gradient can be enhanced by the internal feedback mechanisms that increase *A* with *h*, as we show below.

**Fig 2:**
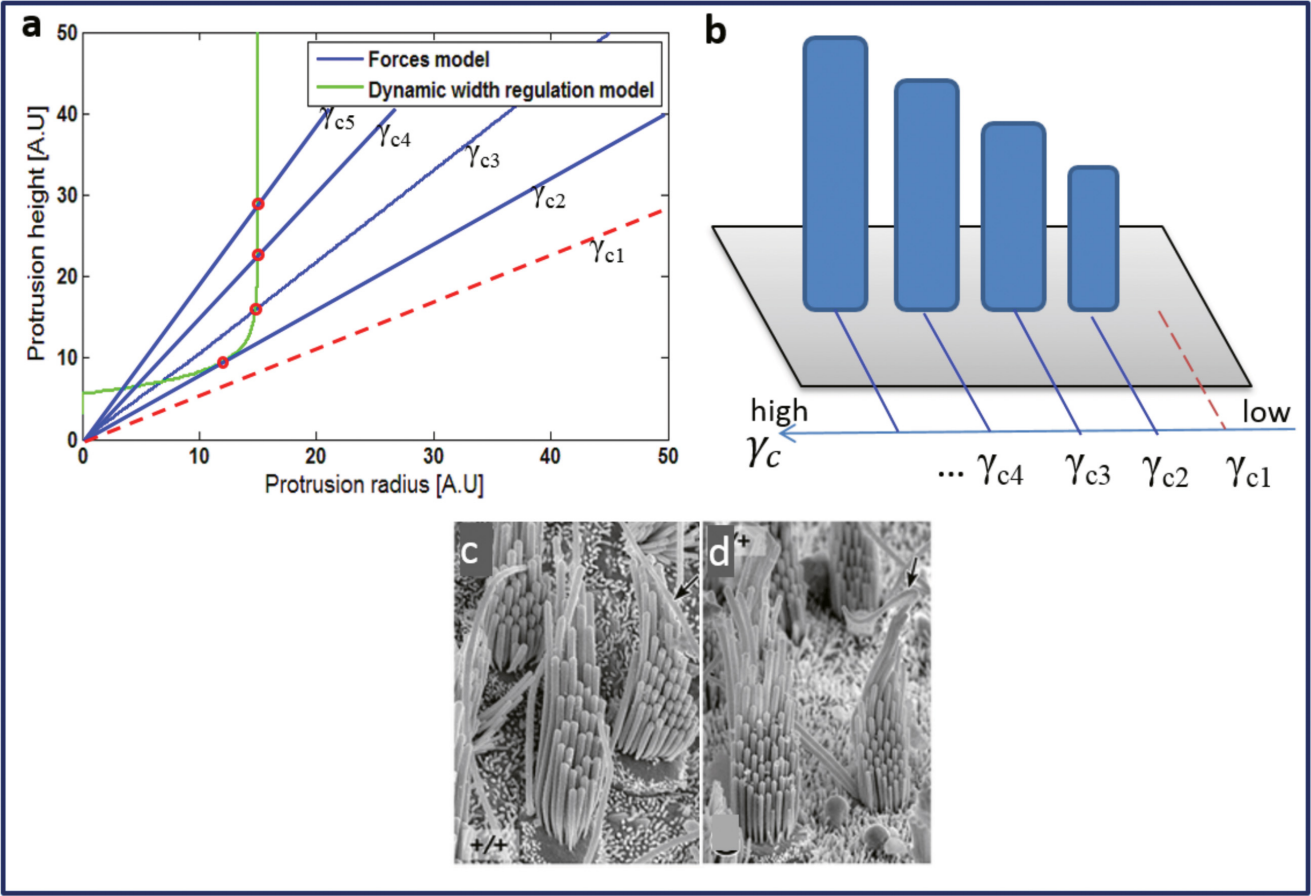
(a) Calculated heights and radii using the theoretical model, with the steady-state solutions indicated by the red circles. Here we take a linear spatial gradient in *γ*_*c*_, and a constant polymerization rate. We get a staircase structure of constant differences in stereocilia heights, but fixed radii (except for the shortest row that are slightly thinner). This result from the model is illustrated in (b). In (c), (d) we compare to the mammalian vestibular stereocilia bundles [26] (Sekerková G et al. (2011) Roles of the espin actin-bundling proteins in the morphogenesis and stabilization of hair cell stereocilia revealed in CBA/CaJ congenic jerker mice. PLoS Genet, 7(3), e1002032-e1002032). The main part of the vestibular bundle has the properties shown in (a,b): stereocilia of equal width (except for thinner first and shortest row), and height increases at a constant gradient between the rows (except for the tallest rows).

Under the assumption of the linear gradient in *γ*_*c*_ the model can account for the SCG and the disappearance of the small MV concomitantly with stereocilia formation, even in the simple case where *A* is independent of *h*, as presented in Fig 2. From the model for the dynamic regulation of *R*_*tip*_ (green line) we find that there is a minimal height for a protrusion, below which its width vanishes. Consider that the viscosity *γ*_*c*_ has the gradient shown in the right panel. For a low value (*γ*_*c*,1_) the St.St height solutions (red line left panel) do not intersect with the St.St width solutions (green line), and there are no stable protrusions. This region corresponds to the apical surface that is free of MV. Higher values of viscosity correspond to stable St.St solutions (blue lines, red circles) of increasing height. The shortest stereocilia are somewhat thinner, but subsequent rows all have the same radius. Both of these features are observed for example in the bullfrog hair cell [23, 24] and in vestibular stereocilia bundles of mammals [25, 26] (Fig 2c and 2d). This analysis yields two results that match previously reported experimental observations: 1) that as the hair cell matures it transforms from a cell with a spatially uniform apical surface covered with relatively homogenous MV, to a non-uniform cell, and 2) beyond a specific location along the apical surface, the MV eventually vanish.

**Fig 3:**
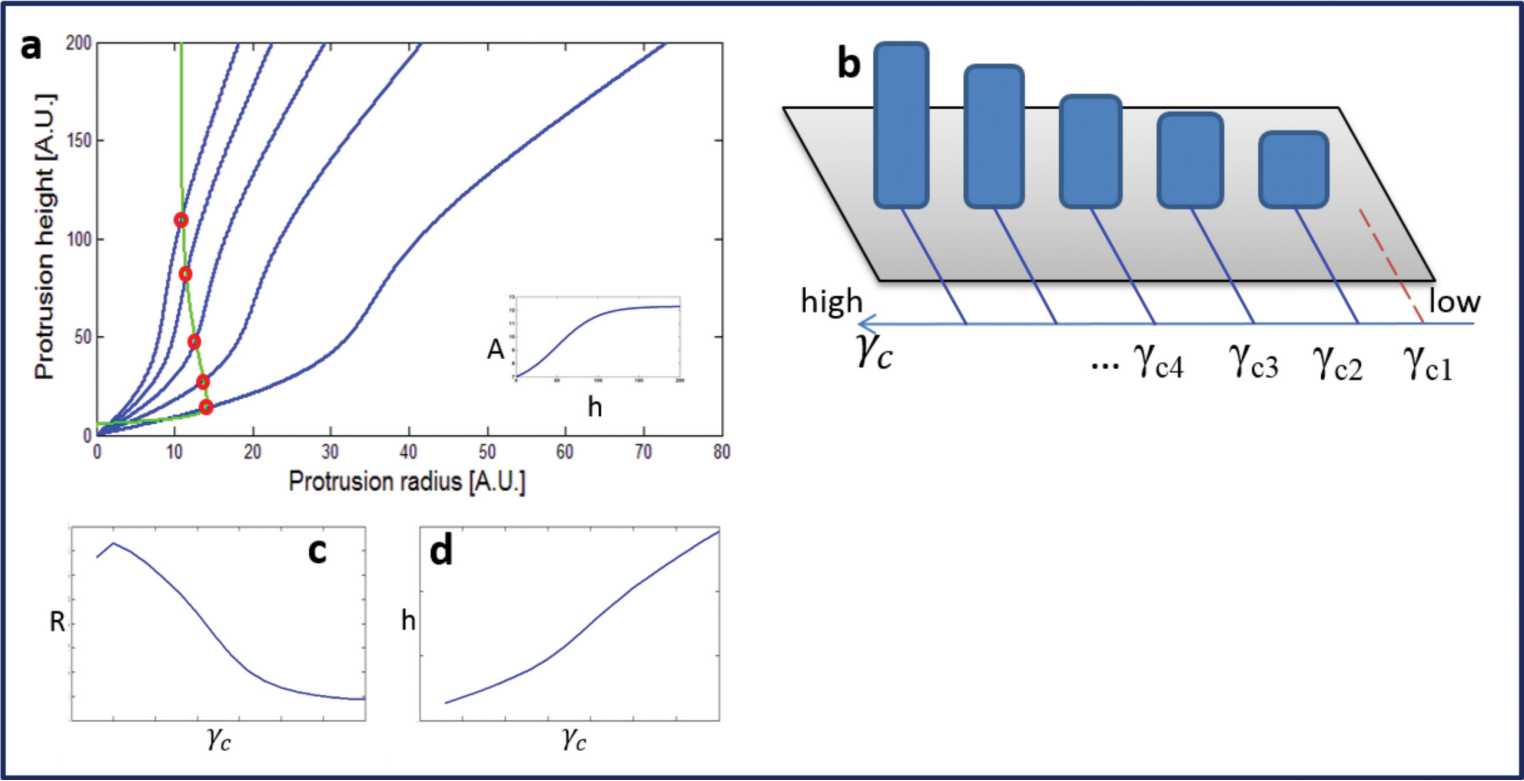
(a) Calculated heights and radii using the theoretical model, with the steady-state solutions indicated by the red circles. Here we take a linear spatial gradient in *γ*_*c*_, and a polymerization rate *A*(*h*) that increases with height (as shown in the inset). We get a staircase structure (b) with radii that become smaller for longer stereocilia (c), and a non-linear ratio in the heights of the rows (d). The non-linear growth of the height and the corresponding decrease in radius [2], are due to the increase in the polymerization rate A(h) with the height.

If the polymerization rate increases with the height *A*(*h*) [13], the staircase heights increase non-linearly (even for a linear gradient in *γ*_*c*_, Eq 2), as indicated in Fig 3. Since the polymerization rate now depends on the height, the radius *R*_*tip*_ differ between stereocilia of different heights. Note that in our model [2] if the rate of actin polymerization and the components of the tip-complex do not have the same height dependence, we get that *R*_*tip*_ decreases with increasing polymerization rate, and therefore decreases with with increasing *h*, as is observed in chick hair cells [27, 28].

**Fig 4:**
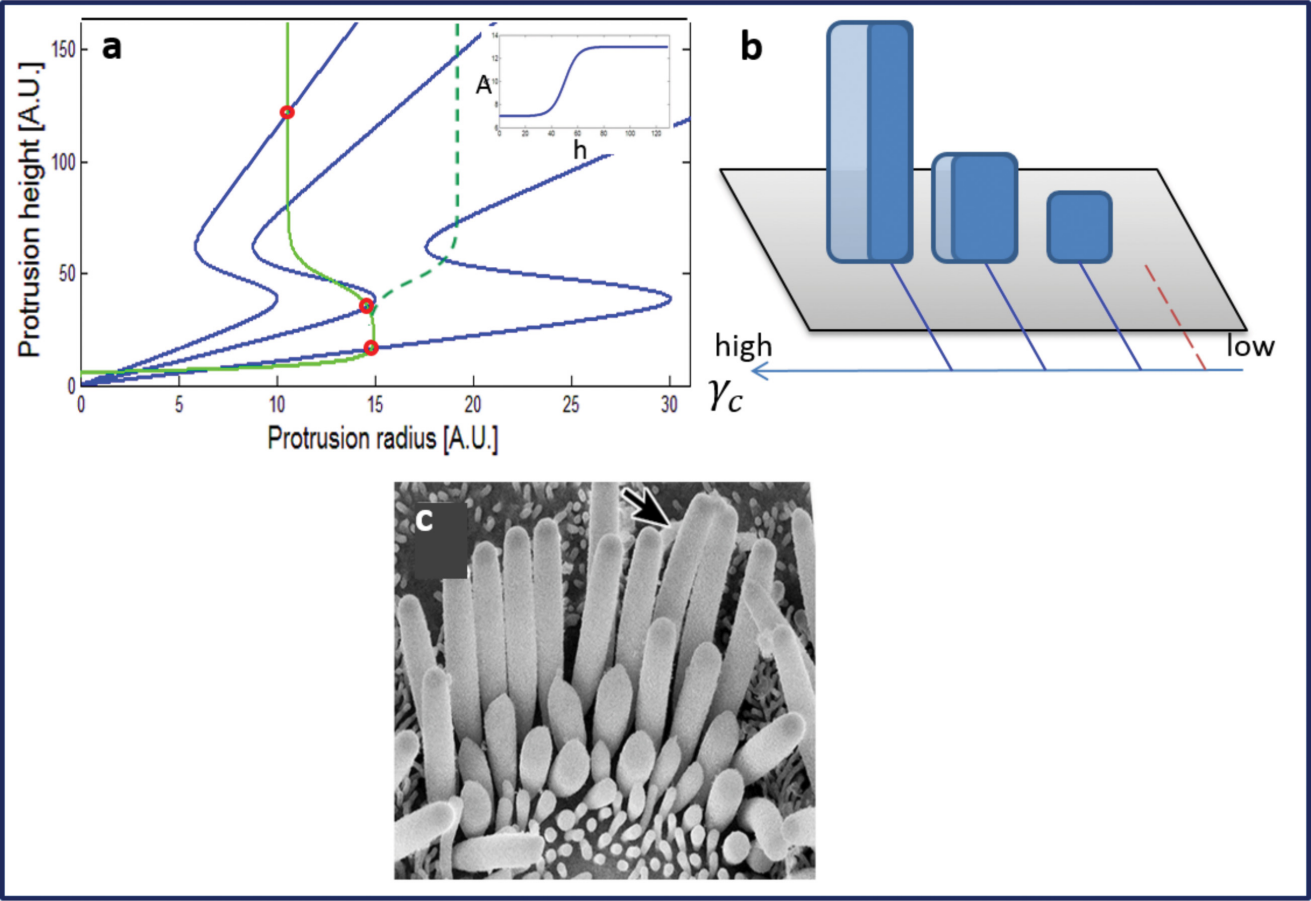
(a) Calculated heights and radii using the theoretical model, with the St.St solutions indicated by the red circles. Here we take a linear spatial gradient in *γ*_*c*_, and a polymerization rates *A*(*h*) that increases sharply with height (as shown in the inset). We get a staircase structure (b) a large jump in height for the first (tallest) row. This row may be either thinner or thicker than the other rows [2], as indicated by the solid green line (a) and dark blue shade in (b) and dashed green line (a) and the light blue shade (b) respectively. This result from the model compares well with the stereocilia bundle of the mammalian inner-hair cell (c) [26] (Sekerková G et al. (2011) Roles of the espin actin-bundling proteins in the morphogenesis and stabilization of hair cell stereocilia revealed in CBA/CaJ congenic jerker mice. PLoS Genet, 7(3), e1002032-e1002032).

When there is a sharp dependence in *A*(*h*), as shown in Fig 4, the first row can be significantly taller than the second row, as is observed in many mammalian inner hair cells [10, 22, 26, 29]. This tallest row may be either thinner or thicker than the second row, depending on the height dependence of the actin polymerization and the flux of the components that compose the tip-complex [2]: *R*_*tip*_ can also increase with the increase in *h*, depending on the regime of the control parameters (see [2] for more details). We note that additional factors could also contribute to the large jump in its height. For example, the existence of proteins associated with the tip-link in all but the first row can affect the polymerization directly through capping activity [15], or indirectly through the difference in Ca^2+^ concentration [30], and both can diminish the heights of the stereocilia except for the first row.

Thus, the model can explain some of the elaborate features of the stereocilia, and link them together. These features include:

1. Transition from a cell completely covered with small and thin MV in its earliest stages into a cell with few rows of thick and long stereocilia arranged in a precise SCG order, as it matures.
2. A large jump of the first row’s height in comparison to the consecutive rows, as observed in inner hair cells of mammals.
3. A relation between the stereocilia radius and its height: In some cases the stereocilia radius is constant regardless of its height [23], while in other cases the longer the stereocilia the thinner it is [27, 28].

### Staircase organization in mutant cells

**Fig 5:**
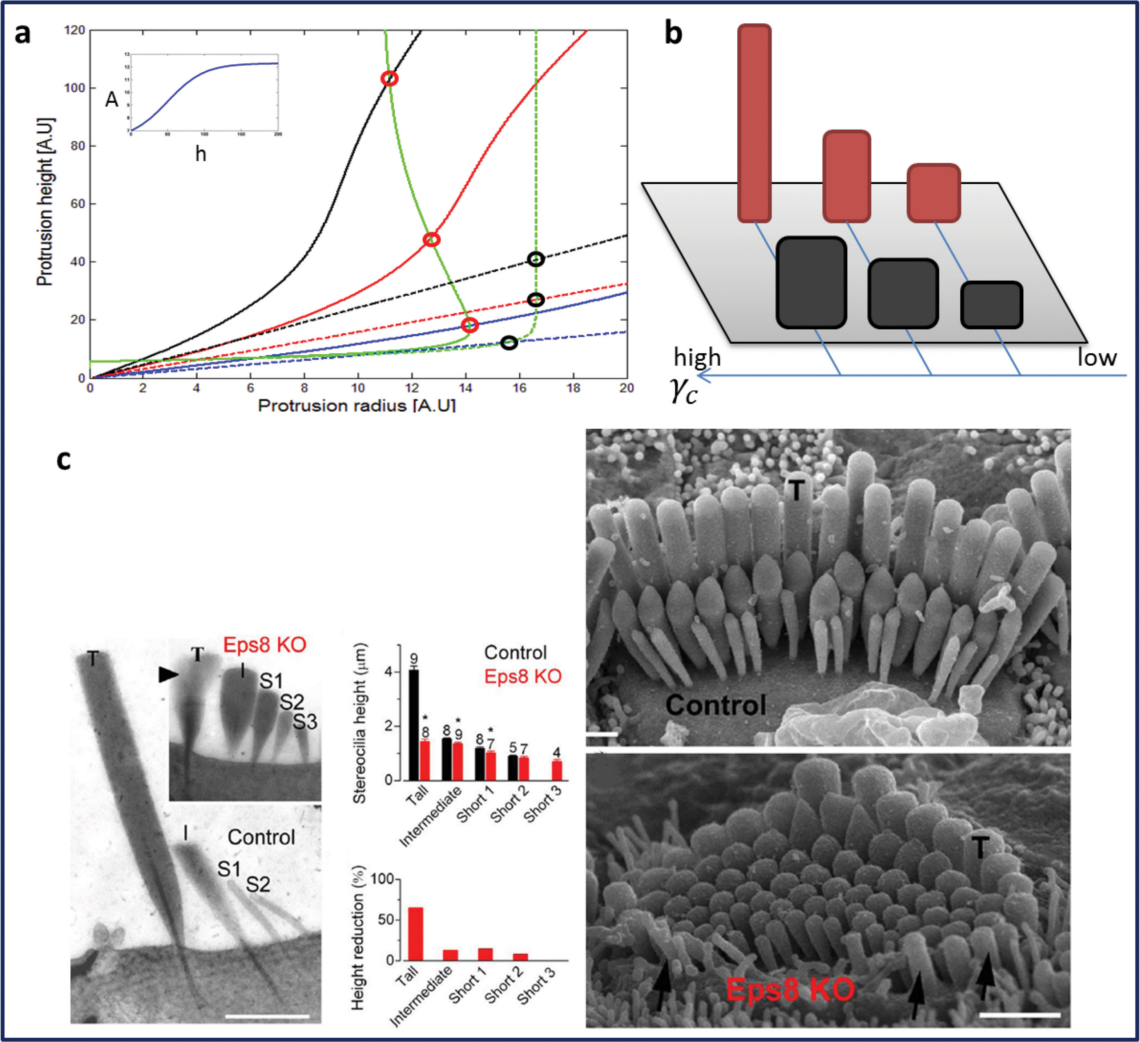
We demonstrate the effect of removal of the height-polymerization feedback, i.e. the increasing relation of *A*(*h*) on *h*. (a) Solid lines show the (normal) condition where *A*(*h*) increases with the height (as shown in the inset), and the stereocilia heights increase non-linearly (black circles, similar to Figs 3 and 4). The dashed lines show the result of reducing the polymerization rate to a constant (independent of the height), resulting in lower and thicker stereocilia (red circles). These two different SCGs are illustrated in (b).(c) Experimental results [32] comparing mammalian (mouse) stereocilia for normal inner-hair-cells, and when Eps8 is knocked out [32] (Zampini V et al. (2011) Eps8 Regulates Hair Bundle Length and Functional Maturation of Mammalian Auditory Hair Cells. PLoS Biol 9(4): e1001048. doi:10.1371/journal.pbio.1001048).

We will now address several outstanding modifications to the stereocilia morphology observed in mutant cells, and attempt to rationalize them according to our model. The first group of mutations involve the myosin-XV, Eps8, and whirlin complex [31]. The loss of any of these components results in short and thick stereocilia, sometimes with a larger number of rows [32, 33]. It is thought that the Eps8-whirlin complex acts at the tips of the stereocilia as a promoter of actin polymerization, and its absence therefore results in a reduction of the polymerization rate. This complex is transported to the stereocilia tips by myosin-XV. Within our model reducing the polymerization rate *A* results in shorter, but thicker protrusions (Fig 5). Since the Eps8-whirlin complex is transported actively (by myosin-XV) to the tips of the stereocilia, it can provide the positive feedback that drives the increase of the polymerization rate with the height [6]. Removal of this feedback component [34] results in a lower and constant *A*, thereby exhibiting no large jumps in the height, only a very shallow and uniform gradient. This residual shallow gradient in these mutants is an indication that there is indeed an overall background spatial gradient in one of the parameters that controls the height, as we have proposed above. Note that while the tallest row shrinks dramatically (Fig 5), the second row is less affected, and the third row remains almost at the same height. Thus, this model explains very well the stereocilia phenotype in mice lacking Eps8 [32] that is otherwise very hard to explain.

Another group of mutations involves the Espin family. Small espin-3 is a cross-linker, and when it is over expressed in MV it results in an increase of the polymerization rate (*A*) by an average factor of ∼ 1.3, a decrease in the severing rate (*β*) by an average factor of ∼ 2, and an overall elongation of the MV by an average factor of 7 [35]. Plugging these numbers in Eq 2, under the assumption that *R*_*tip*_ does not change (it was not measured), results in an increase of the height by a factor of ∼ 3. As a cross-linker, our model predicts that espin acts at the tips of the protrusions to promote the increase in the tip-complex radius, and that this predicted increase in radius results in the observed length increase. Similarly, when espin is missing (Jerker mouse) cochlear stereocilia tend to be much thinner and eventually degrade [26, 36], while when overexpressed [36, 37] the stereocilia are longer and thicker, as predicted from the model for a protein that affect *A*, *β* in the manner described above. Other cross-linkers, such as fascin, have been shown to elongate stereocilia in a similar manner [38].

Another protein found to affect stereocilia structure and long-term stability is Triobp [12], which acts to tightly bundle the actin filaments in the rootlet. In the framework of our model the presence of Triobp can be interpreted as acting to lower the severing rate of the rootlet (*β*), thereby maintaining the rootlet length needed to support the stereocilia (Eq 2). Indeed when Triobp is missing the rootlet does not survive and the stereocilia eventually degrade.

### Effects of inter-stereocilia linkers

So far our model, as expressed in Eq 2, does not include explicitly the effects of the inter-stereocilia linker proteins. These proteins form links between neighboring stereocilia within the SCG, both at the stereocilia base, along its length (side links) and near the tips [39, 40]. These linker proteins are distributed along the stereocilia length by molecular motors that transport them along the actin bundle [41].

There are several effects of inter-stereocilia linkers on the SCG. The most obvious effect is that the linkers act to keep the stereocilia bound to each other, and naturally explains the hexagonal close-packed organization within stereocilia bundles of the type seen in the bullfrog hair cell [16, 23] and in vestibular stereocilia of mammals [25].

Furthermore, such adhesive interactions act to maximize the overall binding and therefore the total contact lengths between neighboring stereocilia. This provides a driving force for stabilizing the SCG (Figure A in S1 Text), with the tall kinocilium providing the anchoring point.

Another effect of inter-stereocilia linkers, especially near the tips of stereocilia within the same row, is to apply an additional restoring force that acts to keep the heights of the stereocilia equal to that of their neighbors. Such a force will therefore act to make the heights much more uniform within each row, making the height distribution much narrower (see further discussion in S1 Text). A similar effect was observed and studied for interacting arrays of MV [42].

## Discussion

We present for the first time a comprehensive biophysical and biochemical model that accounts for the shape (both height and width) of the hair cell stereocilia within the SCG, in both wild-type and mutant cells. The model provides a framework with which to analyze the roles of different proteins inside stereocilia, which are otherwise difficult to determine from experimental data. Our model offers a general framework into which the exact functional forms of the various model parameters, such as *A*(*h*) or *γ*(*x*), can be implemented from experimental measurements once these are made for each particular system. Specifically while we show the possibility for SCG to form in a uniform cell, our results suggest the existence of a gradient in the biochemical and/or mechanical properties of the apical side, such as the cuticular plate, along the planar polarization axis of the hair cell, which cooperates with physical forces and biochemical processes including active transport by myosin motor proteins. While experimental measurements of the biochemical and biophysical properties of this gradient awaits future studies, the presence of planar polarized proteins in inner ear epithelial tissues clearly points towards such a gradient.

## Supporting Information

S1 Text Text file containing figures and further calculations regarding the dynamics and organization of the sterocilia, according to our proposed model. (PDF)
