# Supplementary material for: A Biophysical Model for the Staircase Geometry of Stereocilia
Source: PLoS One. 2015 Jul 24;10(7):e0127926. doi: 10.1371/journal.pone.0127926 (PMC4514777; doi:10.1371/journal.pone.0127926)
Supplement: S1 Text — (PDF) [file pone.0127926.s001.pdf]

Supplementary Material: A biophysical model for the staircase geometry of stereocilia

G. Orly[1], U. Manor[2] and and N. S. Gov[1]*
[1]*Department of Chemical Physics, The Weizmann Institute of Science, P.O. Box 26, Rehovot, Israel 76100*
[2] *Section on Organelle Biology, 35 Convent Drive,*
Porter Neuroscience II, NIH, Bethesda, Md. 20892, U.S.A.

PACS numbers:

From MV to Stereocilia

We here analyze the dynamics of the growth of the stereocilia from the initial small MV that cover the immature hair-cells.

The steady-state height of stereocilia exceed those of the progenitor MV that cover the surface of the immature hair cells, by a factor of $\sim 1-10$ ($\sim 1-100$) for cochlear (vestibular) stereocilia. The polymerization rate is slower in the stereocilia by a factor of ~ 1000 [1] and the radii of stereocilia at the base are typically similar to those of MV. In our model (Eq.2), given these differences in height, the similar base radius and the polymerization rate ratio yields that the ratio γ_c/α for stereocilia should be larger by a factor of $\sim 10^6$ compared to the MV. Since both MV and stereocilia have approximately uniform coverage by some myosin motors (e.g. myosin-I), it is reasonable to assume that the restoring force parameter α is similar in the two systems. We therefore conclude that the increase in height is facilitated by a very dramatic increase in the effective viscosity γ_c below the stereocilia. This calculation may give a causal relationship between the observed formation of the cuticular plate that occurs in conjunction with the height increase of the stereocilia [2].

During the development of the stereocilia from the initial MV, it is sometimes observed that the radius increases in a manner that is not linearly related to the changes in the height [2–4]. In [4] for example, it is observed that while the radius increases by a factor of ~ 2.5 over a period of 12 days, the height increases in a minor way. In our model the radius may increase with the decreasing rate of actin polymerization (as shown in Fig.3), as $R_{tip} \sim 1/A$ [5]. Maintaining a roughly constant height would entail keeping the ratio $A\gamma_c/\beta$ constant (Eq.2), which suggests that the decrease in the polymerization rate is balanced by a larger effective viscosity of the cytoplasmic network (if the severing rate β is unaffected). This compensation can arise if, as suggested above, it coincides with the formation of the cuticular plate.

In mature stereocilia, it was found that the rate of actin polymerization increases with stereocilia height [1]. Similarly, it was found that when actin polymerization was blocked, the rate at which stereocilia height decreased was roughly linear but larger for the taller stereocilia. This can be attributed to the restoring force exerted by the myosin motors along the stereocilia, which is proportional to h, resulting in an exponential retraction which can be approximated to a linear function for short times, before slowing down [5].

While the elongation and shaping of stereocilia clearly depends on actin dynamics, it is possible that at SS conditions the actin core of mature stereocilia becomes static [6]. Our model shows that if the actin polymerization stops the protrusive force F_a vanishes, and therefore in order to maintain the stereocilia shape the underlying support of the stereocilia should similarly become rigid.

INTER-STEREOCILIA LINKERS AND THE SCG

Considering a uniform distribution of inter-stereocilia linkers, it is straight forward that in order to maximize the adhesive interactions between stereocilia of different heights, the stereocilia have to assume the SCG, as opposed to having random locations within the stereocilia bundle. The SCG maximizes the binding energy of the inter-stereocilia linkers. This is illustrated in Fig.A.

RELATION BETWEEN STEREOCILIA HEIGHT AND ROOTLET LENGTH: EVIDENCE FOR THE EFFECTS OF INTER-STEREOCILIA LINKS

We find an experimental manifestation of this additional restoring force in [7], where the relation between the heights and the rootlet lengths of stereocilia was investigated. According to the model [5], the relation between the

height and the rootlet length is given by (Eq.2)

$$h = \frac{\gamma_c}{\alpha}\beta\frac{R_{base}}{R_{tip}}L_{root}\sqrt{1+\left(\frac{L_{root}}{R_{base}}\right)^2} \tag{S1}$$

Since the radius at the base does not change significantly between the first and second rows (and sometimes over all the rows), the rootlet length should give a direct measurement for the ratio A/β. If the severing β is uniform in the cell, then the rootlet gives a measure of the polymerization rate A, which increases with the stereocilia height, in qualitative agreement with previous direct observations [1]. In Fig.B we present the experimental data for the rootlet lengths, given in [7]. The images indicate that between rows of different heights there is indeed a monotonic increasing relation between the height and the rootlet length. However, within each row the height has a much narrower spread compared to the large fluctuations in the rootlet length between neighboring stereocilia.

* Corresponding author: nir.gov@weizmann.ac.il

[1] Rzadzinska AK, Schneider ME, Davies C, Riordan GP, Kachar B. An actin molecular treadmill and myosins maintain stereocilia functional architecture and self-renewal. The Journal of cell biology. 2004;164(6):887–897.
[2] Tilney LG, Tilney MS, Saunders JS, DeRosier DJ. Actin filaments, stereocilia, and hair cells of the bird cochlea: III. The development and differentiation of hair cells and stereocilia. Developmental biology. 1986;116(1):100–118.
[3] Kaltenbach JA, Falzarano PR. Postnatal development of the hamster coclea. I. Growth of hair cells and the organ of Corti. The Journal of comparative neurology. 1994;340(1):87–97.
[4] Rzadzinska A, Schneider M, Noben-Trauth K, Bartles JR, Kachar B. Balanced levels of Espin are critical for stereociliary growth and length maintenance. Cell motility and the cytoskeleton. 2005;62(3):157–165.
[5] Orly G, Naoz M, Gov N. Physical Model for the Geometry of Actin-Based Cellular Protrusions. Biophysical journal. 2014;107(3):576–587.
[6] Zhang DS, Piazza V, Perrin BJ, Rzadzinska AK, Poczatek JC, Wang M, et al. Multi-isotope imaging mass spectrometry reveals slow protein turnover in hair-cell stereocilia. Nature. 2012;481(7382):520–524.
[7] Furness DN, Mahendrasingam S, Ohashi M, Fettiplace R, Hackney CM. The dimensions and composition of stereociliary rootlets in mammalian cochlear hair cells: comparison between high-and low-frequency cells and evidence for a connection to the lateral membrane. The Journal of Neuroscience. 2008;28(25):6342–6353.
[8] Naoz M, Manor U, Sakaguchi H, Kachar B, Gov NS. Protein localization by actin treadmilling and molecular motors regulates stereocilia shape and treadmilling rate. Biophysical journal. 2008;95(12):5706–5718.

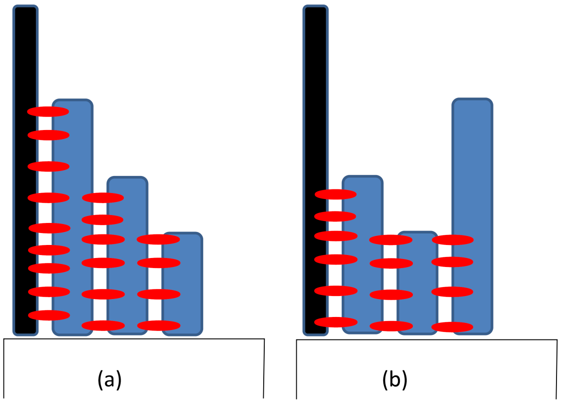

FIG. A: Illustrating the effect of inter-stereocilia linkers (red ovals) in driving the SCG, so that the overall stereocilia binding is maximized. The kinocilium is the tall black bar, shown here to be located at the edge of the apical surface of the cell. The SCG (a) maximizes the contact length between the stereocilia available for linkers, as opposed to a random height organization (b).

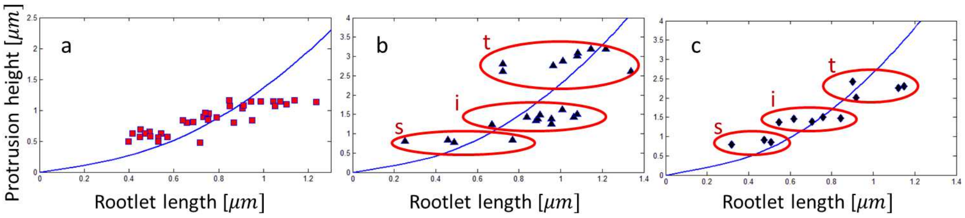

FIG. B: A comparison between the experimental relation of the stereocilia heights (h) and rootlet lengths [7] (symbols) and the theoretical model (Eq.S1, solid lines). In the plots we used for the model calculations the values: $R_{tip} = 0.1\mu m$, $R_{base} = R_{tip}(0.8e^{-2h} + 0.2)$ (based on [8]), and $\beta\,(\gamma_c/\alpha)$ was best fitted to the data. Note that based on our proposal for the origin of the SCG we expect to have a different γ_c for each row (noted by t-tall, i-intermediate and s-short). We plot here the theoretical fit for a constant γ_c, to demonstrate the general expected trend.
